# Analysis of Flavone *C*-Glycosides in the Leaves of *Clinacanthus nutans* (Burm. f.) Lindau by HPTLC and HPLC-UV/DAD

**DOI:** 10.1155/2014/724267

**Published:** 2014-10-22

**Authors:** June Lee Chelyn, Maizatul Hasyima Omar, Nor Syaidatul Akmal Mohd Yousof, Ramesh Ranggasamy, Mohd Isa Wasiman, Zakiah Ismail

**Affiliations:** Phytochemistry Unit, Herbal Medicine Research Centre, Institute for Medical Research, Jalan Pahang, 50588 Kuala Lumpur, Malaysia

## Abstract

*Clinacanthus nutans* (family Acanthaceae) has been used for the treatment of inflammation and herpes viral infection. Currently, there has not been any report on the qualitative and quantitative determination of the chemical markers in the leaves of *C. nutans*. The *C*-glycosidic flavones such as shaftoside, isoorientin, orientin, isovitexin, and vitexin have been found to be major flavonoids in the leaves of this plant. Therefore, we had developed a two-step method using thin-layer chromatography (TLC) and high pressure liquid chromatography (HPLC) for the rapid identification and quantification of the flavones *C*-glycosides in *C. nutans* leaves. The TLC separation of the chemical markers was achieved on silica gel 60 plate using ethyl acetate : formic acid : acetic acid : water (100 : 11 : 11 : 27 v/v/v/v) as the mobile phase. HPLC method was optimized and validated for the quantification of shaftoside, orientin, isovitexin, and vitexin and was shown to be linear in concentration range tested (0.4–200 *μ*g/mL, *r* ^2^ ≥ 0.996), precise (RSD ≤ 4.54%), and accurate (95–105%). The concentration of shaftoside, orientin, vitexin, and isovitexin in *C. nutans* leave samples was 2.55–17.43, 0.00–0.86, 0.00–2.01, and 0.00–0.91 mmol/g, respectively.

## 1. Introduction

*Clinacanthus nutans* (Burm. f.) Lindau (family Acanthaceae) is a medicinal herb that is native to many tropical Asia countries including Malaysia, Thailand, Indonesia, and China [1]. This herb is highly reputed in Thailand where it is known locally as Phaya Yo or Phaya Plong Thong [2]. This herb is also gaining popularity in Malaysia whereby it is more commonly known as Belalai Gajah or Sabah Snake Grass [3]. The fresh leaves of this herb have been traditionally used to treat skin rashes and bites caused by insects and snakes [2, 4]. In addition, the ethanolic extracts from the leaves of* C. nutans* have also been used clinically in the form of topical cream to treat Herpes genitalis and Herpes zoster lesions [5].

Some of the bioactive components that have been isolated from* C. nutans* include the chlorophyll phaeophytin derivatives isolated from the chloroform extract of* C. nutans* leaves and a mixture of cerebrosides and monoacylmonogalactosylglycerol isolated from the ethanolic extract of the fresh leaves of* C. nutans* in which they were shown to inhibit Herpes simplex virus-1 (HSV-1) activity [2, 4]. Other studies have also reported anti-inflammatory and immune-modulatory activities in the ethanolic extracts of* C. nutans* leaves [6, 7]. However, the exact compound or phytochemicals which are responsible for the anti-inflammatory and immune-modulatory activities observed are unknown.

**Figure 1:**
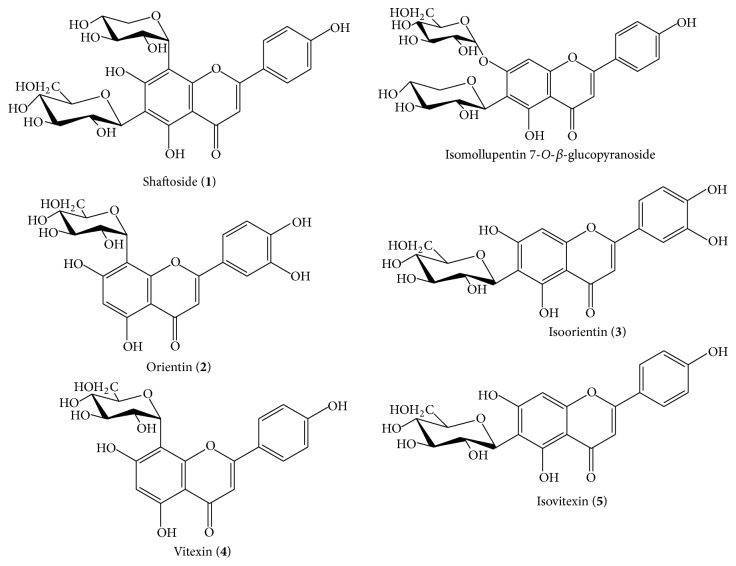
Chemical structures of known flavone* C*-glycosides shaftoside (**1**), isomollupentin 7-*O*-*β*-glucopyranoside, orientin (**2**), isoorientin (**3**), vitexin (**4**), and isovitexin (**5**).

From the known phytochemicals, the six* C-*glycosyl flavones (shown in Figure 1), shaftoside (**1**), isomollupentin 7-*O*-*β*-glucopyranoside, orientin (**2**), isoorientin (**3**), vitexin (**4**), and isovitexin (**5**) isolated from the methanolic extract of* C. nutans* leaves, were of interest due to their rare occurrence in plants [8]. The flavones* C*-glycosides are an important subclass of the flavonoids group of compounds and have been reported in plant from families Verbenaceae, Passifloraceae, Caryophyllaceae, and Polygonaceae [8]. They possess important biological activities including antimicrobial activity (isoorientin, vitexin), hepatoprotective activity (isoorientin), and antioxidant activity (isovitexin) [9–11]. Despite that, there is currently no report on the qualitative and quantitative determination of the chemical markers including the* C*-glycosyl flavones in the leaves of* C. nutans* which hinder the development of* C. nutans* medicinal products. The purpose of this study is therefore to develop a separation technique using high performance thin-layer chromatography (HPTLC) and high performance liquid chromatography coupled to a photodiode array detector (HPLC-UV/DAD) for the simultaneous determination and quantification of flavones* C*-glycosides in* C. nutans* that could accurately identify* C. nutans* raw materials and products.

## 2. Materials and Methods

### 2.1. Plant Materials

Fresh leaves of* C. nutans* were collected from three different geographical locations in Malaysia. The locations that were selected are Taiping, Perak (L1), Kota Tinggi, Johor (L2), and Sendayan, Negeri Sembilan (L3). Voucher specimens UPM/IBS/UB/H39-12, UPM/IBS/UB/H46-12, and SBID 019/12 were prepared and authenticated by botanists from the University Putra Malaysia (UPM) and the Forest Research Institute Malaysia (FRIM). The voucher specimens were deposited in the herbarium of FRIM and Institute for Medical Research.

### 2.2. Chemicals and Standards

Chemical standards shaftoside, isoorientin, orientin, isovitexin, and vitexin (all primary grades) were purchased from Chromadex (Irvine, CA, USA). Solvents acetonitrile and ethanol (both HPLC grade) and ethyl acetate, acetic acid, and formic acid (analytical grade) were purchased from Merck (Merck Co., Darmstadt, Germany). Water was purified with an ultrapure water system (Sartorius, Germany).

### 2.3. Extract and Standard Preparation

The fresh leaves were oven dried at 40°C then grind to fine powder. The ethanolic extract was prepared by adding ethanol (5 mL) to 0.5 g of powdered raw material, sonicated for 30 minutes, and then filtered using Whatman no. 1 filter paper. The supernatant is collected and used for HPTLC and HPLC analysis. The standard stock solution (1 mg/mL) of shaftoside, orientin, isovitexin, and vitexin was prepared in HPLC grade ethanol and stored at 4°C. Working solutions of lower concentration (shaftoside: 20, 40, 60, 80, 100, 150, 175, and 200 *μ*g/mL, orientin, isovitexin, vitexin: 0.2, 0.4, 0.6, 0.8, 10.0, 20, 40, 60, 80, and 100 ug/mL) were prepared by appropriate dilution of the stock solutions in ethanol.

### 2.4. HPTLC Analysis

Thin-layer chromatography was performed on plates precoated with silica gel 60 F254 (Merck, Germany). Samples (5 *μ*L) were applied to the plate as 8 mm width bands using Camag (Switzerland) Linomat V sample applicator. The plate is then developed in an automatic developing chamber (ADC2) (Camag, Switzerland) using ethyl acetate : formic acid : acetic acid : water (100 : 11 :  11 : 27 v/v/v/v) as the mobile phase. Developing distance was 70 mm. After development, the plate was air-dried and derivatized by immersing the plate in natural product reagent (2-aminoethyl diphenylborinate) (1.0 g) in methanol (200 mL) and PEG 400 (10.0 g) in methanol (200 mL).

### 2.5. HPLC Analysis

HPLC-UV/DAD analysis was performed on a Waters Alliance 2695 (Millford, MA, USA) system connected to Waters 2996 photodiode array detector (DAD). The chromatographic separation was performed using a Kinetex PFP column (250 × 4.6 mm, 5 *μ*m, Phenomenex, USA) at 40°C. The solvent system consisted of mixtures of water with 0.8% (v/v) glacial acetic acid (solvent A) and acetonitrile (solvent B). The solvents were degassed before delivering into the system. Samples for HPLC analysis were filtered through a 0.45 *μ*m membrane filter. The optimized HPLC condition is as follows: 5–19% B (30 min), 19–95% B (3 min), 95% B (5 min), 95–5% B (1 min), and 5% B (3 min). The flow rate was 0.7 mL/min. The injection volume was 10 *μ*L. Signal was monitored at 330 nm.

### 2.6. Validation of HPLC Method

The optimized HPLC method was validated in terms of linearity, limit of detection (LOD), limit of quantification (LOQ), precision and accuracy according to the International Conference on Harmonization guidelines [12]. The calibration curves were obtained by the external standard method on eight levels of concentration of standard mixtures, with three injections per level. Calibration curve was plotted using chromatogram peak areas on 330 nm against the known concentrations of standard solutions. A linear regression equation was calculated by least squares method. LOD and LOQ were estimated experimentally by injecting a series of dilute solutions with known concentration until the signal-to-noise ratio reached 3 : 1 for LOD and 10 : 1 for LOQ. Three different concentrations of standard mixtures (5, 10, and 20 *μ*g/mL) were used for intra- and interday precision testing. For intraday precision, the peak area and retention time of three different concentrations of the standard solutions were determined in one day and expressed as relative standard deviation (RSD). For interday precision, the RSD of the standards were determined on three different days. Accuracy of the HPLC method was determined by recovery tests analyzing sample extracts spiked with three different standard mixture concentrations (5, 10, and 20 *μ*g/mL).

## 3. Results and Discussion

### 3.1. HPTLC Screening Results

**Figure 2:**
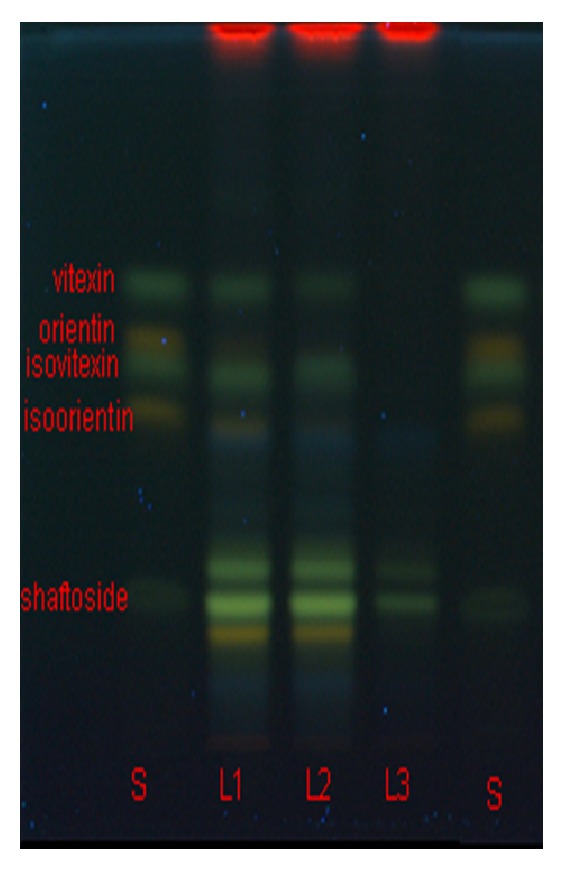
TLC profile of standards and extracts of* C. nutans* leaves from different location. S: standard mixtures of shaftoside (*R*_*f*_ = 0.23), isoorientin (*R*_*f*_ = 0.48), isovitexin (*R*_*f*_ = 0.56), orientin (*R*_*f*_ = 0.60), and vitexin (*R*_*f*_ = 0.66).* C. nutans* samples L1: Taiping, Perak, L2: Kota Tinggi, Johor, and L3: Sendayan, Negeri Sembilan.

TLC chromatographic conditions were optimized to obtain a system in which the flavones* C*-glycosides would be separated in the best manner. The mobile phase containing a mixture of ethyl acetate : formic acid : acetic acid : water (100 : 11 : 11 : 27 v/v/v/v) gave good separation of the compounds of interest. For visualization of the bands, an additional step of derivatization was necessary using natural product reagent followed by PEG 400. The marker compounds after derivatization observed under UV 366 nm gave fluorescent bands on the TLC plate. The flavones* C*-glycosides with the apigenin backbone gave characteristic green coloured bands at retention factors (*R*_*f*_) of 0.23 (shaftoside), 0.56 (isovitexin), and 0.66 (vitexin) while flavones* C*-glycosides with luteolin backbone gave distinct yellow bands for isoorientin and orientin *R*_*f*_ 0.48 and 0.60, respectively. The retention time (*R*_*f*_) and colour of the bands were compared between the standard mixture and samples from three locations to confirm the presence of these markers in samples. The preliminary TLC screening results showed that shaftoside was present in all three sample locations. Isoorientin, isovitexin, orientin, and vitexin were only detected in two locations (L1 and L2) but were absent in the third location (L3) as observed in Figure 2.

### 3.2. Optimization of HPLC Method

**Figure 3:**
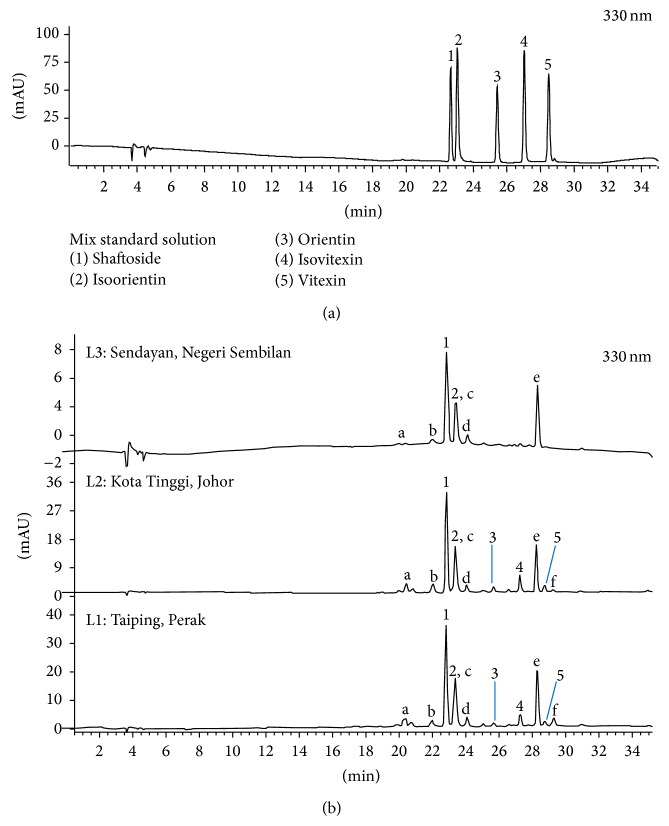
HPLC-UV/DAD chromatogram of (a) standard solution mixture and (b) ethanolic extracts of* C. nutans* leaves, *λ* = 330 nm.* C. nutans* samples L1: Taiping, Perak, L2: Kota Tinggi, Johor, and L3: Sendayan, Negeri Sembilan. (1) Shaftoside, (2) isoorientin, (3) orientin, (4) vitexin, and (5) isovitexin, a–f. Unknown.

Several parameters were optimized to obtain the best HPLC chromatographic separation. The first parameter that was investigated was the use of different mobile phase combination of either water-acetonitrile or water-methanol in which the combination of water-acetonitrile gave better chromatographic resolution compared to the later. Addition of 0.8% glacial acetic acid to the mobile phase was found to improve peak resolution and peak shape and eliminate peak tailing of the marker compounds. Besides that, a gradient elution method improved the resolution of compounds in sample compared to isocratic mode. For this study a shallow gradient profile from 5–19% B for 30 minutes was optimized and used to quantitate markers of interest. In addition, different chromatographic columns including Kinetex PFP column (250 × 4.6 mm, 5 *μ*m, Phenomenex, USA), Sunfire C18 (250 × 4.6 mm, 5 *μ*m, Waters, USA), and Synergi Polar-RP (250 × 4.6 mm, 4 *μ*m, Phenomenex, USA) were tested to improve chromatographic resolution between the markers. A satisfactory separation of marker compounds was obtained using Kinetex PFP column (250 × 4.6 mm, 5 *μ*m, Phenomenex, USA). However, investigation of the peak purity of isoorientin in samples showed that the isoorientin peak (**2**) was coeluting with another compound (Peak c) as shown in Figure 3(b). Therefore, quantification of isoorientin was not carried out in this study. The HPLC separation was monitored at 330 nm according to the absorption maxima of the marker compounds. The HPLC-UV/DAD chromatogram of the standard solution mixture and the ethanolic extracts of* C. nutans* leaves at 330 nm are shown in Figures 3(a) and 3(b), respectively.

### 3.3. HPLC Validation Data

The linear range, regression equation, correlation coefficient of each marker, and LOD and LOQ values are summarized in Table 1. The eight point calibration curve showed good linearity (*R* ^2^ = 0.996) in the given concentration ranges. The LOD and LOQ values showed the adequate sensitivity of the HPLC method developed (Table 1). The interday and intraday precision range from 0.62 to 4.54% (Table 2) showed that the method was repeatable. The recovery values of the marker compounds are presented in Table 2. The average recoveries which ranged from 95.33% to 105.13% showed good accuracy of the method. However, recoveries were above 100% for certain samples possibly due to the inaccuracy of spiking at lower ranges. The compounds that showed high recoveries were orientin 105% (amount recovered 5.25 *μ*g/mL), shaftoside 102% (10.21 *μ*g/mL), isovitexin 101% (5.09 *μ*g/mL), and vitexin 102% and 101% (5.1 *μ*g/mL and 10.15 *μ*g/mL).

### 3.4. Sample Analysis

The optimized and validated method was applied to determine the concentration of the marker compounds in the samples from the three geographical locations. The content of shaftoside, orientin, isovitexin, and vitexin in samples is presented in Table 3. The results showed that shaftoside was the major flavone present in samples from all three locations in which concentration ranges from 2.55 mmol/g to 17.43 mmol/g. The other known flavones* C*-glycosides were present in lower amounts, ranging from the highest for isovitexin (0.00–2.01 mmol/g), followed by orientin (0.00–0.86 mmol/g), to the lowest amount which was vitexin (0.00–0.91 mmol/g). Flavones* C*-glycosides are favourable chemical markers due to their large structural diversity that could help to distinguish rapidly between other plant species [13]. The results also allowed us to conclude that shaftoside would be best suited as chemical markers for* C. nutans* raw material as it is found in all locations in the highest quantity. The lower amount of flavones detected including shaftoside in L3 compared to L1 and L2 could be caused by differences in climate, soil regime of the different region, and differences in processing such as harvest time, drying, and storage all of which may affect the composition of flavonoids.

## 4. Conclusion

Chemical markers are essential as part of identification and also quality control of herbal material. In the present study, HPTLC technique was used for the rapid identification of five known flavones* C*-glycosides in* C. nutans* raw materials. In HPTLC, the unique characteristic fluorescent bands after derivatization provided important clues for the identification of the major flavone present in the samples. A second step using HPLC-UV/DAD technique was employed for the simultaneous detection and quantification of these known flavonoids. The HPLC-UV/DAD method developed in this present study was effective, accurate, precise, and sensitive for the quantification of four known flavones* C*-glycosides (shaftoside, orientin, isovitexin, and vitexin) of interest. The method was also applied to identify and quantify the chemical markers of interest in crude powdered leaves of* C. nutans* from three geographical regions in Malaysia. From the present study, the compound shaftoside could be recommended as a marker for* C. nutans* raw material as it is present in all locations and is also available as a commercial standard for method validation. The current method developed in this study is also useful for the evaluation of quality of* C. nutans* raw materials and its commercial products.

## Figures and Tables

**Table 1:** Linear range, regression equations, LOD, and LOQ for quantitative analysis of HPLC (*n* = 3).

Compound	*T*_*R*_ (min)	*λ* (nm)	Linear range (*µ*g/mL)	Regression equation^a^	*R* ^2^	LOD (*µ*g/mL)	LOQ (*µ*g/mL)
Shaftoside	22.79	271, 337	20.0–200	*y* = 28370*x* − 230484	*R* ^2^ = 0.999	0.2	0.6
Orientin	25.58	255, 349	0.8–100	*y* = 27655*x* − 27061	*R* ^2^ = 0.996	0.4	0.8
Isovitexin	27.16	269, 337	0.8–100	*y* = 34067*x* + 47328	*R* ^2^ = 0.999	0.4	0.8
Vitexin	28.61	268, 337	0.8–100	*y* = 29217*x* + 33931	*R* ^2^ = 0.997	0.4	0.8

^a^ *y* = *ax* + *b*, where *x* is concentration in *µ*g/mL and *y* is area under curve at UV 330 nm wavelength.

**Table 2:** Intra- and interday precision, recovery, and accuracy data.

Compound	Precision (RSD % RT, AUC)			Recovery		
	Concentration (*µ*g/mL)	Intraday (*n* = 3)	Interday (*n* = 6)	Amount added (*µ*g/mL)	Recovery (%)	RSD (%)
Shaftoside	5	3.24	2.80	5	95.33	2.18
	10	2.96	2.11	10	102.10	3.49
	20	0.85	3.39	20	95.50	0.74

Orientin	5	3.24	2.94	5	105.13	1.43
	10	2.96	1.92	10	99.53	1.03
	20	0.85	3.08	20	98.11	0.10

Isovitexin	5	1.55	0.96	5	101.97	2.93
	10	4.18	2.43	10	97.67	3.53
	20	0.70	3.29	20	96.49	0.53

Vitexin	5	2.38	2.11	5	102.61	1.25
	10	3.11	4.54	10	101.50	2.10
	20	0.62	3.48	20	95.52	1.43

AUC: area under curve.

**Table 3:** Content of shaftoside, orientin, isovitexin, and vitexin in samples of *C. nutans* from different locations. L1: Taiping, Perak, L2: Kota Tinggi, Johor, and L3: Sendayan, Negeri Sembilan.

Compound	Content of marker compounds (mmol/g)		
	L1	L2	L3
Shaftoside	17.43 ± 0.01	16.33 ± 0.03	2.55 ± 0.04
Orientin	0.86 ± 0.04	0.59 ± 0.003	ND
Isovitexin	2.01 ± 0.02	1.03 ± 0.09	ND
Vitexin	0.91 ± 0.03	0.43 ± 0.006	ND

ND: not detected.

Values are means ± SD, *n* = 3.
